# Sequential Biotherapy Targeting IL-5 and IL-4/13 in Patients with Eosinophilic Asthma with Sinusitis and Otitis Media

**DOI:** 10.3390/biom12040522

**Published:** 2022-03-30

**Authors:** Ayumi Chikumoto, Keiji Oishi, Kazuki Hamada, Tsunahiko Hirano, Tomoyuki Kakugawa, Keiko Kanesada, Kazuto Matsunaga

**Affiliations:** 1 Department of Respiratory Medicine and Infectious Disease, Graduate School of Medicine, Yamaguchi University, Ube 755-8505, Japan; chiku05@yamaguchi-u.ac.jp (A.C.); khamada@yamaguchi-u.ac.jp (K.H.); tsuna@yamaguchi-u.ac.jp (T.H.); 2 Department of Medicine and Clinical Science, Graduate School of Medicine, Yamaguchi University, Ube 755-8505, Japan; ohishk@yamaguchi-u.ac.jp; 3 Department of Pulmonary and Gerontology, Graduate School of Medicine, Yamaguchi University, Ube 755-8505, Japan; tomoyukikakugawa@gmail.com; 4 Nonohana Clinic, Yamaguchi 753-0221, Japan; keiko33@mocha.ocn.ne.jp

**Keywords:** sequential biotherapy, anti-IL-5 receptor antibody, anti-IL-4/13 receptor monoclonal antibody, multiple Type 2 inflammatory pathways, eosinophil sinusitis, eosinophilia otitis media

## Abstract

Type 2 (T2) inflammation plays an important role in the pathogenesis of allergic diseases such as asthma, eosinophilic chronic rhinosinusitis (ECRS), or eosinophilic otitis media (EOM). Currently, in severe asthma with the T2 phenotype, biologics targeting mediators of T2 inflammation dramatically improve the management of severe asthma. While treatment with a single biologic is common, little is known about cases of the sequential use of two biologics. Here, we report a case of severe asthma with refractory ECRS and EOM in which total control of these allergic diseases could not be achieved with a single biologic but could be achieved via the sequential use of the anti-IL-5 receptor antibody and human anti-IL-4/13 receptor monoclonal antibody. It is suggested that it is necessary to control multiple T2 inflammatory pathways to achieve total control of severe allergic diseases. Sequential biotherapy may help solve the clinical challenges associated with single-agent molecular-targeted therapies.

## 1. Introduction

Type 2 (T2) inflammation plays an important role in the pathogenesis of allergic diseases such as asthma, eosinophilic chronic rhinosinusitis (ECRS), chronic rhinosinusitis with nasal polyps (CRSwNP), and eosinophilic otitis media (EOM) [1,2,3]. Recently, in severe asthma with the T2 phenotype, biologics targeting mediators of T2 inflammation dramatically improved the management of severe asthma. Currently available biologics for severe asthma include the anti-IgE antibody, anti-IL-5 antibody, anti-IL-5 receptor antibody, and human anti-IL-4/13 receptor monoclonal antibody [4]. It is common to treat the condition with a single biologic, and it is selected according to biomarkers, uncontrolled clinical problems, comorbidities, etc. Little is known about cases of the sequential use of two biologics, and there is only one report from Yasuba et al. [5].

Herein, we report a case in which solving the clinical problem was difficult with a single biologic but was possible with the sequential use of the anti-IL-5 receptor antibody and human anti-IL-4/13 receptor monoclonal antibody. Sequential biotherapy solved the control of airway inflammation, symptoms, and exacerbation of asthma and improved the complications of refractory ECRS and EOM.

## 2. Case Report

**Figure 1:**
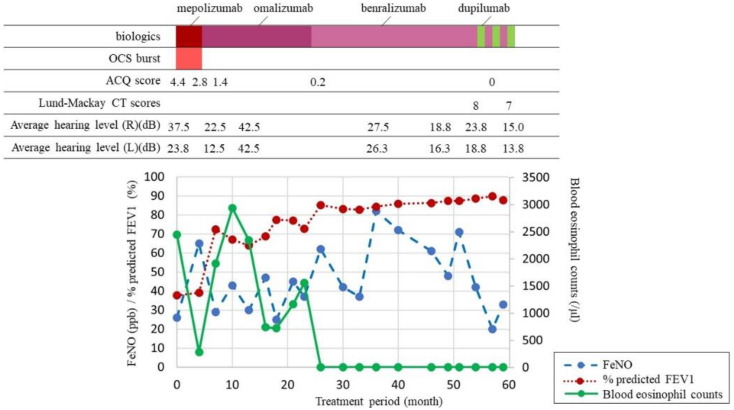
Treatment course of patient.

**Figure 2:**
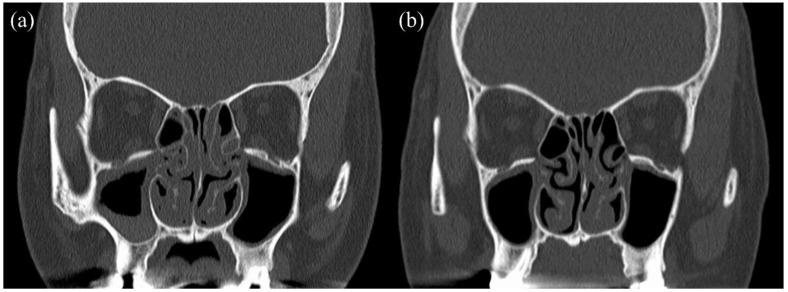
Sinus CT before and after sequential biotherapy: (**a**) before sequential biotherapy, (**b**) after sequential biotherapy for 7 months.

Herein, we describe the case of a 41-year-old woman who was referred for treatment of severe asthma. She was a non-smoker. She had been receiving one puff of vilanterol trifenatate 25 μg and fluticasone furoate 200 μg once daily and two puffs of tiotropium bromide hydrate 2.5 μg once daily. She was also taking montelukast sodium (10 mg daily). As stage lighting staff, she had to miss work once a month due to asthma exacerbation, and she used oral corticosteroids (OCS) for each asthma exacerbation. She reported the loss of her sense of smell, dizziness, and otorrhea, as well as complicated ECRS and EOM. Her EOM was diagnosed when she was 33 years old. She underwent multiple bilateral myringotomy and tympanostomy tubes due to severe EOM. The clinical findings at the first visit were as follows: % predicted FEV1, 37.7%; FeNO, 26 ppb; blood eosinophil count, 2441 cells/μL; total IgE level, 657 IU/mL. Her specific IgE levels for vernal grass, dactylis, Japanese cedar, cypress, and Aspergillus were positive. The ACQ score at that time was 4.4 points. The patient’s treatment course is shown in Figure 1. First, mepolizumab, which is a monoclonal anti-IL-5 antibody, was initiated for severe eosinophil asthma. Three months later, she was no longer absent and her ACQ improved to 2.8 points, but the need for OCS due to frequent exacerbations remained. Thus, we considered that mepolizumab had insufficient therapeutic efficacy and started with omalizumab, an anti-IgE antibody. Two months later, the asthma exacerbation decreased, OCS was no longer necessary, and the % predicted FEV1 improved to 85.2%. However, the loss of her sense of smell, dizziness, and otorrhea caused by ECRS and EOM persisted, and she still had trouble working. Thereafter, these symptoms did not improve, and the blood eosinophil count increased. Nineteen months after the initiation of omalizumab, we switched to benralizumab, which is an anti-IL-5 receptor alpha antibody. During the third month after the initiation of benralizumab, her blood eosinophil counts decreased to 0 cells/μL, and her ACQ improved to 0.2 points. Although we continued treatment with benralizumab for approximately 25 months, her otorrhea, dizziness, and loss of sense of smell worsened. Especially for EOM, there was no improvement in her hearing level, and the therapeutic efficacy was poor. We hypothesized that sequential biologic therapy with benralizumab as anti-IL-5 therapy and dupilumab, which inhibits IL-4 and IL-13 signaling, would have a significant effect in this patient based on the following reasons: (i) the inadequate response to treatment with mepolizumab and omalizumab, (ii) the effects of treatment of asthma with benralizumab, (iii) prolonged high FeNO levels, and (iv) promising data on therapeutic response to dupilumab for ECRS. Therefore, we initiated sequential biotherapy, which comprised a cycle of dupilumab administration four times every 2 weeks for 2 months after a single administration of benralizumab per month for 2 months. Sequential biotherapy was initiated not only to maintain asthma control but also to improve the sense of smell and otorrhea and suppress the progression of ECRS and EOM. Subsequently, asthma control remained well maintained, and the sense of smell, dizziness, hearing level, and otorrhea improved. Moreover, she no longer had any problems with work. Figure 2 shows a sinus CT before and after sequential biotherapy. After sequential biotherapy for 7 months, improvements in sinus shadowing were observed. The ACQ at that time was 0 points, and % predicted FEV1 was 89.9%. She is still undergoing sequential biotherapy.

## 3. Discussion

In this case report, we present a patient with severe asthma with refractory ECRS and EOM in which total control of these allergic diseases could not be achieved with a single biologic but could be achieved with sequential biotherapy. In addition, sequential biotherapy has the potential to simultaneously control a variety of T2 airway inflammations.

First, in severe asthma with the coexistence of other allergic diseases, total control of allergic diseases may not be achieved by single-agent molecular-targeted therapy, and it has been shown that sequential biotherapy may be effective in such cases. In this case, several treatments with single-agent biologics were attempted. However, total control of asthma and multiple allergic diseases was not achieved. Previously, a randomized, double-blind, placebo-controlled trial assessed the efficacy and safety of mepolizumab in patients with severe nasal polyposis [6]. A total of 105 patients with chronic sinusitis with recurrent eosinophil nasal polyps requiring surgery were divided into a mepolizumab group (*n* = 54) and a placebo group (*n* = 51) for 25 weeks. The number of patients who did not require surgery was higher in the mepolizumab group than in the placebo group (16 (30%) vs. 5 (10%); *p* = 0.006). It has been reported that mepolizumab treatment significantly improves nasal symptom scores. Thus, although there were a reasonable number of cases where CRSwNP could be controlled with mepolizumab alone, surgery was necessary for 70% of patients even after treatment with mepolizumab.

In contrast, dupilumab is likely to be effective in treating severe asthma with CRSwNP. There are also multicenter, randomized, double-blind, parallel-group studies assessing dupilumab in adult patients with severe CRSwNP (LIBERTY NP SINUS-24 and LIBERTY NP SINUS-52) [7]. Dupilumab reduced polyp size, sinus opacification, and the severity of symptoms in both studies. Furthermore, CRSwNP is characterized by intense edema or the formation of pseudocysts filled with plasma proteins [2]. It has been reported that IL-4- and IL-13-dependent T2 inflammation downregulates t-PA expression and induces excessive fibrin deposition through the suppression of fibrinolysis [8,9]. This may be one of the reasons why this patient had improved ECRS control with dupilumab.

However, the adverse event of blood eosinophilia is often a problem with dupilumab. Blood eosinophilia with clinical symptoms has been reported as an adverse event in SINUS-24 and SINUS-52 (0.9%). In the LIVERTY Asthma QUEST, which was a randomized controlled trial to evaluate dupilumab efficacy and safety in patients with uncontrolled moderate-to-severe asthma, blood eosinophilia (≥2000) has been reported (about 9%) [10]. Based on this knowledge, dupilumab should be administered with caution in patients with high blood eosinophil levels. Our patient also had a high blood eosinophil count of more than 2000 cells/μL before the administration of the anti-IL-5 antibody and during the use of the anti-IgE antibody. Therefore, we concluded that the continuous use of dupilumab would be risky. Sequential biotherapy may be safe, even in cases with initially high blood eosinophil counts.

Asthma and CRSwNP share a common pathophysiology of T2 inflammation. T2 inflammation-driven cytokines, such as IL-4, IL-5, and IL-13, are associated with systemic and airway inflammation [11]. Moreover, group 2 innate lymphoid cells are recognized as key controllers of T2 inflammation [12]. Cluster analysis in patients with CRS confirmed that the presence of T2 inflammation was highly associated with CRSwNP and asthma [13]. We also reported that sinus surgery affected asthma control through the suppression of airway/systemic T2 inflammation in asthma patients with CRSwNP [14]. In our sequential biotherapy, it is speculated that airway inflammation was controlled by simultaneously suppressing these different T2 inflammation pathways, which led to the improved management of complications.

Second, sequential biotherapy has the potential to control airway inflammation. Blood eosinophils are commonly used to monitor systemic T2 inflammation, which is strongly triggered by IL-5 [15]. FeNO is a useful surrogate marker for assessing airway inflammation, which is primarily triggered by IL-4 and IL-13 in the bronchial mucosa [16]. We have previously reported the response to high-dose systemic corticosteroids in terms of asthma control, lung function, blood eosinophils, and FeNO in patients with severe asthma [17]. There was variability in their responses to systemic corticosteroids in blood eosinophils and FeNO, and it was essential to suppress both T2 biomarkers to improve asthma control [18]. In addition, a previous study examined the effect of inhibiting multiple cytokines involved in asthma in a mouse model using a bispecific antibody [19]. They generated monospecific and bispecific antibodies that target IL-4Rα and IL-5. The monospecific antibodies suppressed eosinophilia and/or IgE synthesis but not goblet cell metaplasia (GCM) and bronchoalveolar hyperreactivity (BHR). On the other hand, only the administration of the IL-4Rα/IL-5 bispecific antibody and the combination of the monospecific antibody of IL-4Rα and IL-5 inhibited GCM and BHR. Bispecific antibodies targeting multiple cytokine-signaling pathways may be effective against asthma phenotypes with difficult-to-treat GCM. In our case, it was difficult to evaluate the inhibitory effect of GCM because mucus plug formation was not noticeable on the chest CT. In addition, pathological evaluations and airway hypersensitivity assessments were not performed in this case. However, to control multiple allergic diseases associated with T2 inflammation, multiple pathways of T2 inflammation need to be controlled. In this case, the anti-IL-5 receptor antibody and human anti-IL-4/13 receptor monoclonal antibody were used alternately, and different T2 inflammatory pathways controlled severe airway inflammation. These results are presumed to significantly improve asthma control. Recently, Yasuba et al. reported the use of cycling therapy with benralizumab and dupilumab for severe eosinophilic asthma with ECRS and EOM. Our sequential biotherapy targeted a similar patient and was poorly controlled with a single biologic. In the present case, FeNO and blood eosinophil counts were strongly controlled, and symptom control and airflow limitation were also markedly improved, which reinforced the effectiveness of the sequential use of the two biologics. It should also be noted that no adverse events, such as a worsening of symptoms, pulmonary function, or T2 inflammation, were observed in this patient when switching from one biologic to another. As a limitation of our present case, the possibility that long-term biologics prior to sequential biotherapy may have affected the treatment results cannot be completely ruled out. However, we propose the consideration of sequential biotherapy as the next therapeutic strategy when single-agent biologics is not effective.

## 4. Conclusions

Sequential biotherapy may help solve the clinical challenges associated with single-agent molecular-targeted therapies. Further studies should be conducted to determine whether sequential biotherapy can contribute to the achievement of multiple responses in patients with severe asthma who cannot achieve total control of their allergic disease.

## Data Availability

Data sharing is not applicable to this article.
